# Commentary on Notification and Recordkeeping of Occupational Mesothelioma in India

**DOI:** 10.1002/ajim.70060

**Published:** 2026-02-07

**Authors:** Raja Singh, Arthur L. Frank

**Affiliations:** ^1^ Department of Environmental and Occupational Health, Dornsife School of Public Health Drexel University Philadelphia Pennsylvania USA

**Keywords:** asbestos‐related diseases, cancer registry, factories regulator, mines regulator, occupational health, pleural mesothelioma

## Abstract

In India, some occupational diseases are notifiable under the Mines Act, 1952, and the Factories Act, 1948. Mesothelioma, primarily attributable to asbestos exposure, has been listed specifically as one of the notifiable diseases under the Mines Act, 1952, and is notifiable under the category of occupational cancer in the Factories Act, 1948. The total number of cases of mesothelioma notified to the Directorate General of Mines Safety under the mining safety law was zero from 2004 to 2024. Similarly, under the factory safety law, only one case of occupational cancer was notified in one state, in a country of 28 states and 8 union territories (mesothelioma being listed under occupational cancer and not a separate entry under the factories law). This is in sharp contrast to the medical literature, where a large number of cases have been published by researchers and doctors from Indian hospitals. The absence of notified disease may not automatically mean the absence of disease. Further, a parallel National Cancer Registry Program, which is not only for occupational cancers, but which may overlap with occupational cases, covers only 16% of the country's population. With clear lack of notification of cases and underreporting of occupational mesothelioma, and cancer not being declared as universally notifiable at the national level, disease surveillance in India may need to be invigorated so that easily preventable disease is reduced, load on the already strained healthcare infrastructure is decreased, and overall national medical costs are reduced in the future.

## 1 Indian Occupational Disease Notification

The Indian parliament legislated the Occupational Safety, Health, and Working Conditions Code in 2020 [1]. This legislation subsumed 13 existing major labor laws in India including the two major laws, the Factories Act, 1948, and the Mines Act, 1952 [2, 3]. Major portions of these two laws have been retained in the new Code including the provision for the notification of occupational diseases. Under Section 89 of the Factories Act, 1948, it is a legal requirement for the manager of the factory or the medical practitioner attending to a worker in the factory to report to the Chief Inspector of Factories any worker who has contracted any disease listed in the Third Schedule of the Factories Act, 1948.

The same is the case with the Mines Act, 1952, in which Section 25 states that the owner, agent/manager, or any medical practitioner attending to a person has to report to the Chief Inspector of Mines if any person employed in the mine contracts a “disease connected with mining operations” as listed on a schedule by the central government. Under the new code, however, the list of notifiable diseases from the two laws have been merged into a common list in the Third Schedule.

It must also be noted that there is a difference between the health and safety regulation of factories versus mines. The primary difference is that factory safety falls under the purview of the state governments, and the central government (the Indian federal government) merely provides guidance through the Directorate General of Factories Advice Service and Labor Institutes or DGFASLI, whereas mine safety is directly under the purview of the central government, which appoints a Chief Inspector of Mines at the national level. The Directorate General of Mines Safety, Dhanbad, has been designated by the Government of India as the Chief Inspector under the Mines Act, 1952 [4].

The intent of notification of disease implies that occupational health is taken seriously, by first endeavoring to obtain an accurate count of cases, and thus enable the reduction of a preventable burden of disease [5]. It also makes economic sense, as the disease burden can in the future reduce the value generated by the workers and employees [6]. But hardly any studies have actually addressed occupational diseases notification by medical professionals and which diseases have been recorded by the regulators. There have also been calls for having a separate occupational registry in India [7]. The legal definition and jurisdiction of what is an occupational disease in a factory may differ from an occupational disease in a mine. A mine involves extraction from the ground, whereas further processing and milling of minerals may be in the jurisdiction of factories [8]. In the process of jurisdiction division, the true case incidence should be reported and recorded. Silicosis has been reportable under the Factories Act as well as the Mines Law, which indicates its status as a hazard in both extraction and processing. Similarly, mesothelioma, which is reportable as such under the mining law, is reportable as an occupational cancer under the Factories Law.

In India, apart from the occupational disease recordkeeping under the factory and the mining laws, there is also the National Cancer Registry Program or NCRP [9]. Notable is the possible overlap of all those occupational cancers which can be notified under the two laws (mining and factories), but may also be recorded under the NCRP if the worker approaches a hospital under the NCRP as are many tertiary‐level care hospitals. Even though occupational cancer is already notifiable under the factory and the mine labor laws, cancer as a disease is not widely reported in India. Notification of diseases is linked to its recordkeeping, as without a legal mandate to notify, hospitals may not be incentivised to share these data with the authorities. Wider cancer notification in India is being opposed by the Indian Health Ministry despite recommendations by the Indian Council of Medical Research‐National Centre for Disease Informatics and Research or ICMR‐NCDIR [9]. Robust recordkeeping of cancer may be weakened due to a lack of a legal mandate to report cancer cases, which can be done by making cancer notifiable in India [10]. This lack of legal mandate also means that the NCRP remains voluntary for hospitals, and the data from the NCRP may not tell the complete picture.

## 2 Overview of Asbestos and Its Occupational Effect in India

India is also a founding member of the International Labour Organization (ILO). India has not ratified the ILO Occupational Cancer Convention, 1974. But there is another important convention that India has not ratified, the Asbestos Convention, 1986 [11, 12]. Mesothelioma, which is a rare malignancy, can be considered a surrogate for exposure to asbestos of all six types, including chrysotile [13]. Mesothelioma, in particular, is of grave concern as India continues to be a major importer, processor, and user of asbestos [14, 15, 16]. In response to civil society and judicial action, in 1986 India stopped asbestos mining, and a similar order was issued in 1993 [17]. The DGFASLI has studied the health effects of occupational use of asbestos [18]. The study did not include the investigation of any mesothelioma cases, and these details were not available in the report [19]. Coverage of malignancies with long latency periods, from 15 to 40 years, was also not available in this report, which did not include retired workers in the study. The study may have covered some relevant areas of investigation, but surely lacks detail on the incidence of mesothelioma and investigation of the long‐term latency of disease that is caused by exposure to asbestos. This exclusion, in spirit, is not in line with the order of the Supreme Court of India, which had directed all asbestos industries to “maintain and keep maintaining the health record of every worker” up to a minimum period of 40 years from the beginning of employment. It is also possible that industries may simply keep healthy workers and fire the unhealthy ones (or coerce them to resign), leading to a “healthy worker effect” [20].

## 3 The Recordkeeping of Mesothelioma in India

The central government, under the Mines Act, 1952, has declared “cancer of the lung or the stomach or the pleura and the peritoneum (i.e., mesothelioma)” as reportable/notifiable diseases since 1986. Further, in the case of the Factories Act, 1948, the “Third Schedule” lists 29 notifiable diseases, which includes cancer of the skin separately and has an entry for “Occupational Cancer” separately under which mesothelioma is supposed to be notified.

Studies by other researchers in two hospitals in Rajasthan and Gujarat have reported 76 cases (2015–2020) and 126 cases (2015–2019) of mesothelioma, respectively [21, 22]. In the first study, all patients denied any past exposure to asbestos, but 91% of the patients had a history of mining, direct or indirect, in marble and granite mines or quarries. Marble from certain Indian regions has tremolite contamination. This also means that some of these reported cases are likely occupational mesotheliomas, which should have been reported under the Mines Act, 1952.

Data from another study of Indian mesothelioma case numbers are unclear on whether these cases are from occupational or nonoccupational exposure [23]. Therefore, there exists a gap and the number of mesotheliomas which can be typically classified as occupational have not been compiled before. An understanding of this may be vital to inform policy and to allocate already legislated responsibility to various stakeholders in the management of occupational diseases in India. Further, determining mesothelioma case numbers from occupational settings will be the first step to quantify and to inform about the likely causative factors of asbestos exposure, and will help ascertain whether the source is occupational or nonoccupational (e.g., exposure to asbestos in a factory, or disguised exposure through working in a marble cutting unit). This is important as asbestos is still widely imported in India, used in India, processed in Indian factories, used by the Indian population, and disposed of into the general environment [14, 24, 25, 26]. There are also identified cases of other minerals, including talc, which have asbestos as a contaminant to which the Indian population is exposed in occupational and nonoccupational settings, without awareness or precautions [8, 27, 28, 29].

Apart from the mechanism of recording and notification of occupational mesotheliomas through the Mines and Factory Regulator, there is also reporting through the NCRP, as stated before. In a previous study, the authors of this commentary found 1126 cases in a period of 4 years (2012–2016) [23]. The NCRP reported 54 cases during the same time period [9]. This is because, despite being operational since 1981, the cancer registry program has been able to cover only 16% of the Indian population [10, 30]. In this study mentioned above, it was found that only 25% of the hospitals in the study were part of the NCRP, which further highlights the lack of coverage of the NCRP.

## 4 Number of Notified Occupational Mesothelioma Cases in India

As shown above, it is important to first ascertain the number of occupational mesothelioma cases that have been recorded and notified in India. A time period of 20 years from 2004 to 2024 was selected, and an information request under the Right to Information Act, 2005, was filed to request data on occupational mesotheliomas under the Mines Act 1952 from the DGMS, to which mine management and medical practitioners from across the country must report and notify occupational diseases. The same process of requesting information was repeated with the Chief Inspectors of Factories of all the applicable Indian states and union territories to whom factory management and physicians must report cases, 27 of which provided a response. The DGFASLI, which is a central Government of India organization under the Ministry of Labor and Employment was also requested for information since the internal statistics cell of the DGFASLI also collects disease notification information from the Chief Inspectors of Factories of individual states and publishes this information annually as part of the Standard Reference Note [31]. It was found that the Standard Reference Notes were available on the website only from 2006 to 2023 (data for 2005–2023), but in these reports the compilation of occupational diseases was only made from the year 2016 onwards, which means the data are only available from 2015 until 2022.

In the time period between 2004 and 2024, the DGMS stated that no single case of mesothelioma was reported to the Chief Inspector of Mines from throughout India.

Further, the data from 2015 to 2022, from the DGFASLI, also had no cases of occupational cancer shared by the Chief Inspector of Factories to the DGFASLI [31]. The data received from 27 states and union territories have been compiled in Table 1. Out of the 27, three denied the information, one state was not eligible to provide information (as there was no enforcement of the Factories Act, 1948 in Sikkim), and three states reported zero factories in the state. None of the states reported an occupational cancer. Only one state, Haryana, reported one case of occupational cancer, and Maharashtra reported a single case of bladder cancer, whilst not classifying it directly as “occupational cancer” but mentioning it separately.

**Table 1:** The data from the Chief Inspector of Factories from 27 Indian states and union territories reporting occupational cancers from 2004 to 2024, along with other information.

S. no.	State/Union Territory name	No. of registered factories in the state/Union Territory	Number of occupational cancers notified to the Chief Inspector of Factories from 2004 to 2024	Remarks
1	National Capital Territory of Delhi	8661	0	Source: Provided through letter ID No. 25/RTI/CIF/LAB/2024/48 dated January 21st, 2025.
2	Goa	Denied	Denied	Reply given as “Information does not exist” Source: Provided through letter ref No. RTI/460/IFB/2025/3723 dated January 17th, 2025.
3	Himachal Pradesh	3013	0	Source: Provided through letter No. L&E(Fac)(RTI)/2024(R.S) dated January 9th, 2025.
4	Odisha	5209	0	Source: Provided through letter no. DOFB‐RTI CASE‐0003‐2025‐87 dated January 7th, 2025.
5	Gujarat	40,495	Denied	Stated that information is “not available” Source: Provided through letter no. Dish/Sankalan/RTI/73/2024/73 dated January 20th, 2025.
6	Meghalaya	363	0	Source: Provided through letter No. FBM‐132/2007/Pt‐II/34 dated January 15th, 2025.
7	Madhya Pradesh	Not provided (Denied)	0	‘निरंक’ (in Hindi, translated as “Nil”). Source: Provided through letter no. 23/RTI/2024/2597 dated December 27th, 2024.
8	Tamil Nadu	52,138	0	The State provided date of other diseases such as silicosis, talc silicosis etc. Source: Provided through letter no. F3/00619/2025 dated January 21st, 2025.
9	Maharashtra	38,879	0	5 cases of asbestosis (2012‐2; 2014‐1; 2015‐1; 2023‐1) and bladder cancer notified, but mesothelioma not notified. Source: Provided through letter no. DISH/Estt/491/2 dated January 24th, 2025.
10	Tripura	0	0	Source: Provided through letter no. F.2(323)‐FB/ESTT/RTI/2011/Vol‐II/1806‐07 dated January 18th, 2025.
11	Assam	10,223	0	Source: Provided through letter no. E‐599050 dated January 29th, 2025.
12	Andhra Pradesh	24,642	0	Source: Provided through letter no. LAE05/32/2025‐A SEC‐DOF dated January 27th, 2025.
13	Nagaland	43	0	Source: Provided through letter no. LBR/TECH‐365/2005(Vol‐VI)(Pt)/31 dated January 29th, 2025.
14	Sikkim	N.A.	N.A	Not Applicable as Factories Act 1948 is yet to be enforced in the state of Sikkim. Source: Provided through letter no. 370/LD/Adm dated January 29th, 2025.
15	Rajasthan	11,226	0	Source: Provided through letter no. PIO/2025/92 dated January 29th, 2025.
16	Lakshadweep	0	0	Source: Provided through letter no. 36/9/2011‐EEL/13 dated January 11th, 2024.
17	Manipur	404	0	Source: Provided through letter no. W‐2/IND/2021‐RTI(Pt)/117 dated January 29th, 2025.
18	Mizoram	0	0	Source: Provided through letter no. F.23012/1/2023‐LESDE dated January 17th, 2025.
19	West Bengal	Denied	Denied	Information not provided by either the Labor department or the Directorate of Factories.
20	Telangana	—	0	Silicosis cases have been reported. Source: Provided through letter no. A3/RTI Act/5393/2024 dated January 18th, 2025. Information on total number of factories not requested.
21	Punjab	11,378	0	Source: Provided through letter no. RTI/2025/I/1036966/2025 dated February 18th, 2025.
22	Uttar Pradesh	—	0	Uttar Pradesh, 18 district responses were sought. Total number of factories not requested. District‐wise replies received.
23	Haryana	9501	1	1 case from Faridabad. Other districts reported other notified diseases. Source: Provided through letter no. RTI/02/FS/2024/157 dated January 14th, 2025. And No. 645 same date.
24	Kerala	22,539	0	Source: Provided through letter no. DFB/3234/2024‐G4 dated January 20th, 2025.
25	Puducherry	3155	0	Source: Provided through letter no. 4100/CIF&B/B3/RTI/2024 dated January 13th, 2025.
26	Andaman and Nicobar Islands	39	0	Source: Provided through reply in RTI no. UALET/R/E/24/00005 dated January 7th, 2025.
27	Jharkhand	—	—	No provided due to a technical issue.

*Note:* Source of the Information is reply letters from the Directors of factories (or equivalents) of states. In replies, whenever a “nil” reply was provides, it has been interpreted as value “0.” The replies are in the form of correspondences. *Source:* Chief Inspector of Factories of States and Union Territories or equivalents.

Research papers from academic institutions have reported mesothelioma cases in India [21, 22, 23, 32, 33, 34, 35]. In other cases where research papers have been published, the medical records section of the same hospital denies any case within that same time period [34, 36]. It is concerning that mesotheliomas have been diagnosed and reported at large hospitals, but these are not necessarily reported to the factory or the mine regulators. This can be interpreted to either mean that the mesotheliomas recorded in India by other studies are completely from nonoccupational exposure to asbestos, or secondly, that despite the cases of occupational mesothelioma suggested by research papers, the lack of reporting to the factory and mine regulators means that there is an absence of recordkeeping and a violation of the provisions of the two laws. Such underreporting of notifiable diseases is not uncommon in medical settings.

## 5 Recommendations for Occupational Mesothelioma Notification and Recordkeeping

We recommend that, first, the provisions in both laws where medical practitioners are penalized for failure to report occupational diseases must be more strictly implemented. Nonreporting by doctors, despite their moral and legal responsibility, could be avoided by the levy of fines. Medical practitioners must be made aware during their education and during their practice of their duty to notify agencies of occupational disease cases. The National Medical Commission should take up this cause and create mechanisms to make doctors aware of their responsibility to report occupational diseases. Second, the Chief Inspector of Mines at the central level and the Chief Inspector of Factories at the state level must create infrastructure and mechanisms so that doctors are constantly reminded of their duty, and there is ease of reporting. There should be regular outreach programs at all hospitals, especially tertiary care facilities and referral centers, by these authorities. Third, the possibility of conflict of interest at the level of states, where factories and mines are wealth generators and may not be regulated in spirit for being revenue creators, must be re‐evaluated from the perspective of social and financial cost of an unhealthy workforce. The healthcare cost due to occupational diseases must be quantified, and this must be subtracted from the sum total of revenue generated from factories and mines. Fourth, there should be coordination between the NCRP run by the Indian Council of Medical Research‐National Centre for Disease Informatics Research and the recordkeeping done by the mining and factory regulators. Instances where there are cases recorded for an area of a cancer, such as mesothelioma, by the NCRP but not by the mine regulator decrease confidence in laws and their enforcement and should be avoided. Fifth, cancer must be made notifiable across the country, despite it being a noncommunicable disease. Seventeen states in India have made cancer notifiable in line with ICMR‐NCDIR recommendations [10]. This will enable universality in reporting and prevent underreporting, especially in cases where there is no clear‐cut differentiation between occupational and nonoccupational aetiology, which can also be recorded. Sixth, mesothelioma has been a separately‐classified occupational disease under the Mines Act, 1952, since the notification in 1986, but after the promulgation of the Occupational Safety, Health and Working Conditions Code, 2020, it has not been retained in the list of notifiable diseases in the Third Schedule. While other mine‐related diseases, such as coal workers’ pneumoconiosis, seem to be retained, mesothelioma is absent in the new list. It seems to have been merged, as in the Factories Act, 1948, with the broader class of occupational cancer. As a rare malignancy associated with a specific exposure to asbestos, it should be listed as a separate entry in the Third Schedule of the new Occupational Safety, Health and Working Conditions Code, 2020. Seventh, a separate occupational disease registry, or better still, a mesothelioma registry, should be created specifically across all of India. This should include surveillance of former workers and miners to take into account the long latency of asbestos‐related diseases like mesothelioma. Lastly, once notified, mesothelioma cases must be made part of a surveillance system, which leads to analysis, interpretation, and dissemination of the findings for action to prevent mesothelioma. In general, India must focus on notification and proper reporting of occupational mesothelioma, and cancer should be declared universally notifiable as a disease. Disease surveillance in India may need to be invigorated so that easily preventable disease is better dealt with and reduces the load on the healthcare system.

## Author Contributions

**Raja Singh:** conceptualization, data curation, investigation, methodology, project administration, resources, writing – original draft, writing – review and editing. **Arthur L. Frank:** project administration, resources, supervision, validation, writing – review and editing.

## Funding

The authors received no specific funding for this work.

## Ethics Statement

This work did not involve any human participants, or human tissue, and used only nonidentifiable/without human identifier data available in the public domain and hence is not under the purview of ethics clearance. It should be further noted that the information released under the Right to Information Act, 2005, is information in the public domain. Further, it implies that the information is nonpersonal and does not have any human identifier, as public authorities by law can only release nonpersonal information [37].

## Conflicts of Interest

The first author declares no conflict of interest. The second author conducts medical‐legal work on asbestos, primarily for plaintiffs.

## Data Availability

All the data have been reported in the manuscript itself.
